# Ethyl 2-benzyl-3-[3-(4-chloro­phen­yl)-1-phenyl-1*H*-pyrazol-4-yl]-4,6-dioxo-5-phenyl­octa­hydro­pyrrolo­[3,4-*c*]pyrrole-1-carboxyl­ate

**DOI:** 10.1107/S1600536812002450

**Published:** 2012-01-31

**Authors:** P. Kamatchi, G. Jagadeesan, M. Pramesh, P. T. Perumal, S. Aravindhan

**Affiliations:** a Department of Chemistry, Bharathi Women’s College, Chennai 600 108, India; b Department of Physics, Presidency College, Chennai 600 005, India; c Organic Chemistry Laboratory, CLRI, Chennai 600 020, India

## Abstract

The title compound, C_37_H_31_ClN_4_O_4_, crystallizes with two mol­ecules (*A* and *B*) in the asymmetric unit. The pyrrole rings in both mol­ecules are connected *via cis* fusion, whereas one ring has a twisted conformation and the other assumes a half-chair conformation. In the crystal, the *A* mol­ecules form inversion dimers *via* a pair of C—H⋯Cl inter­actions, while the *B* mol­ecules form chains propagating in [1
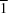
0], *via* C—H⋯O inter­actions. In the crystal, there are also a number of C—H⋯π inter­actions present.

## Related literature

For the bioactivity of pyrazole derivatives, see: Sullivan *et al.* (2006 ▶); Patel *et al.* (2010 ▶); Siu *et al.* (2008 ▶). For conformation studies, see: Nardelli (1983 ▶).
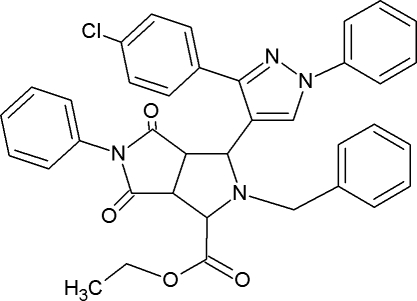


## Experimental

### 

#### Crystal data

C_37_H_31_ClN_4_O_4_
*M* *_r_* = 631.11
Triclinic, 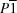

*a* = 12.8293 (8) Å
*b* = 13.3467 (8) Å
*c* = 22.0754 (11) Å
α = 83.897 (4)°
β = 81.585 (5)°
γ = 62.213 (6)°
*V* = 3304.9 (3) Å^3^
*Z* = 4
Mo *K*α radiation
μ = 0.16 mm^−1^
*T* = 293 K
0.2 × 0.2 × 0.2 mm

#### Data collection

Oxford Diffraction Xcalibur Eos diffractometer
Absorption correction: multi-scan (*CrysAlis PRO*; Oxford Diffraction, 2009 ▶) *T*_min_ = 0.978, *T*_max_ = 0.984
29939 measured reflections
15365 independent reflections
7600 reflections with *I* > 2σ(*I*)
*R*_int_ = 0.027

#### Refinement

*R*[*F* ^2^ > 2σ(*F* ^2^)] = 0.067
*wR*(*F* ^2^) = 0.225
*S* = 1.00
15365 reflections
831 parameters
259 restraints
H-atom parameters constrained
Δρ_max_ = 0.88 e Å^−3^
Δρ_min_ = −0.57 e Å^−3^

### 

Data collection: *CrysAlis PRO* (Oxford Diffraction, 2009 ▶); cell refinement: *CrysAlis PRO*; data reduction: *CrysAlis PRO*; program(s) used to solve structure: *SHELXS97* (Sheldrick, 2008 ▶); program(s) used to refine structure: *SHELXL97* (Sheldrick, 2008 ▶); molecular graphics: *PLATON* (Spek, 2009 ▶); software used to prepare material for publication: *PLATON* and *publCIF* (Westrip, 2010 ▶).

## Supplementary Material

Crystal structure: contains datablock(s) I, global. DOI: 10.1107/S1600536812002450/su2345sup1.cif

Structure factors: contains datablock(s) I. DOI: 10.1107/S1600536812002450/su2345Isup2.hkl

Supplementary material file. DOI: 10.1107/S1600536812002450/su2345Isup3.cml

Additional supplementary materials:  crystallographic information; 3D view; checkCIF report

## Figures and Tables

**Table 1:** Hydrogen-bond geometry (Å, °) *Cg*1, *Cg*2 and *Cg*3 are the centroids of the C10–C15, C27′–C32′ and C33′–C38′ rings, respectively.

*D*—H⋯*A*	*D*—H	H⋯*A*	*D*⋯*A*	*D*—H⋯*A*
C7—H7⋯O44′	0.98	2.45	3.311 (3)	146
C42′—H42*A*⋯Cl1′^i^	0.96	2.76	3.707 (10)	170
C14′—H14′⋯*Cg*1	0.93	2.79	3.653 (4)	154
C17′—H17′⋯*Cg*2^ii^	0.93	2.95	3.738 (4)	143
C29—H29⋯*Cg*1^iii^	0.93	2.87	3.633 (3)	140
C42′—H42*B*⋯*Cg*3^iv^	0.96	2.80	3.866 (9)	153

Symmetry codes: (i) 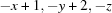; (ii) 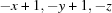; (iii) 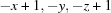; (iv) 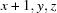.

**Fig. 1.**
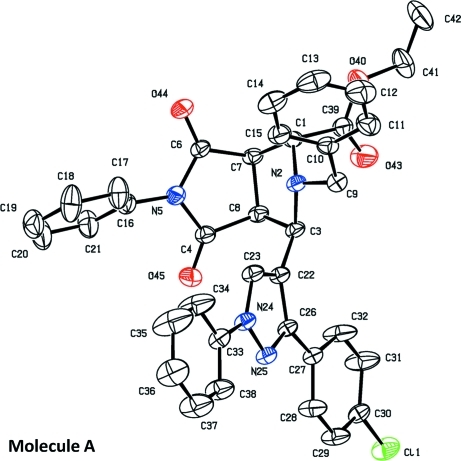
The molecular structure of one of the two independent molecules (A) of the title compound, showing 30% probability displacement ellipsoids and the atom numbering scheme [H atoms have been omitted for clarity].

**Fig. 2.**
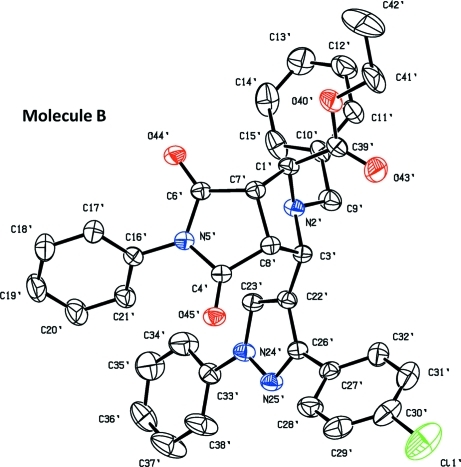
The molecular structure of the other independent molecules (B) of the title compound, showing 30% probability displacement ellipsoids and the atom numbering scheme [H atoms have been omitted for clarity].

**Fig. 3.**
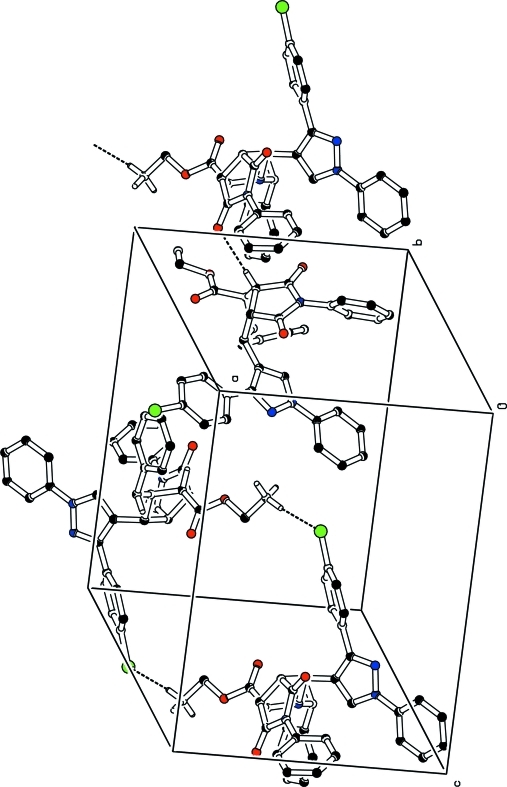
A partial view of the crystal packing of the title compound. H atoms not involved in the C—H···O and C—H···.Cl interactions (dashed lines) have been omitted for clarity.

## supplementary crystallographic information

### Comment

Pyrazoles exhibit a variety of pharmacological properties for e.g antibacterial and anti-inflammatory activities (Sullivan *et al.*, 2006; Patel *et al.*, 2010). One of the pyrazole derivatives shows nucleosidase inhibitory activity against Staphylococcus aureus (Siu *et al.*, 2008). In view of their importance, the crystal structure determination of the title compound was carried out and the results are presented herein.

The molecular structure of the two independent molecules (A and B) of the title compound are shown in Figs. 1 and 2, respectively. In each molecule the pyrazole rings, (N24,N25,C26,C22,C23) in A and (N24',N25',C26',C22',C23') in B, are planar. In molecule A it is almost coplanar with the chlorophenyl ring attached to C26 as well as the phenyl ring attached at N24, with dihedral angles of 3.91 (16) and 4.88 (17) °, respectively. However, in molecule B the pyrazole ring is inclined to the chlorophenyl ring by 48.71 (17) °, and by 5.42 (18) ° to the phenyl ring at N24'.

The torsion angle H8—C8—C7—H7 in molecule A is 12.23 °, and H8'-C8'-C7'-H7' in molecule B is -14.81 °, which defines the ring fusion in the pyrrolo-pyrrole moieties as *cis*.

The pyrrole rings [(N5,C6—C8,C4) in A, and (N5',C6'-C8',C4') in B] assume half-chair (or envelope) conformations, with atoms C8 and C8' at the flap in molecules A and B, respectively, whereas the other pyrrole rings [(N2,C1,C7,C8,C3) in A and (N2',C1',C7',C8',C3') in B] have twisted conformations: defined by the asymmetry parameters (Nardelli, 1983), DS (N2) = 0.086 (2) Å and D2 (C8) = 0.010 (1) Å in molecule A, and DS (C3') = 0.018 (3) and D2 (C8') = 0.073 (2) in molecule B.

The partial double bond character of bonds C6—N5 [1.386 (3) Å] and C6'-N5' [1.390 (4) Å], and N5—C4 [1.395 (3) Å] and N5'-C4' [1.393 (3) Å], shows a high degree of electron delocalization.

In the crystal, the A molecules form inversion dimers *via* a pair of C—H···Cl interactions, while the B molecules form chains, propagating in [1 - 1 0], *via* C—H···O interactions (Fig. 3 and Table 1). There are also a number of C—H···π interactions present (Table 1).

### Experimental

A mixture of pyrazole aldehyde (0.3 g), benzylethylglycinate (0.177 g) and maleimide (0.158 g) was refluxed in toluene(15 ml) until completion of the reaction as evidenced by TLC analysis. The solvent was evaporated under reduced pressure. The crude product was purified by column chromatography on silica gel [Merck, 100–200 mesh, ethylacetate–petroleum ether (10:90)] to afford pure product. Crystals, suitable for X-ray analysis, where obtained by slow evaporation of a solution in ethylacetate.

### Refinement

The NH H-atom was located in a difference electron-density map and was freely refined. The C-bound H-atoms were included in calculated positions and treated as riding atoms: C—H = 0.95, 0.98, 0.99 and 1.00 Å for CH(aromatic), CH_3_, CH_2_ and CH(methine) H-atoms, respectively, with *U*_iso_(H) = k × *U*_eq_(parent C-atom), where k = 1.5 for CH_3_ H-atoms and k = 1.2 for all other H-atoms. A potential solvent accessible void of 135.8 Å^3^ was detected but no residual electon density could be located in the final difference Fourier map.

### Crystal data

**Table:**

C_37_H_31_ClN_4_O_4_	*V* = 3304.9 (3) Å^3^
*M**_r_* = 631.11	*Z* = 4
Triclinic, *P*1	*F*(000) = 1320
Hall symbol: -P 1	*D*_x_ = 1.268 Mg m^−^^3^
*a* = 12.8293 (8) Å	Mo *K*α radiation, λ = 0.71073 Å
*b* = 13.3467 (8) Å	θ = 3.0–29.3°
*c* = 22.0754 (11) Å	µ = 0.16 mm^−^^1^
α = 83.897 (4)°	*T* = 293 K
β = 81.585 (5)°	Triclinic, colourless
γ = 62.213 (6)°	0.2 × 0.2 × 0.2 mm

### Data collection

**Table:**

Oxford Diffraction Xcalibur Eos diffractometer	15365 independent reflections
Radiation source: fine-focus sealed tube	7600 reflections with *I* > 2σ(*I*)
graphite	*R*_int_ = 0.027
Detector resolution: 15.9821 pixels mm^-1^	θ_max_ = 29.3°, θ_min_ = 2.8°
ω scans	*h* = −17→17
Absorption correction: multi-scan (CrysAlis PRO; Oxford Diffraction, 2009)	*k* = −17→18
*T*_min_ = 0.978, *T*_max_ = 0.984	*l* = −29→27
29939 measured reflections	

### Refinement

**Table:**

Refinement on *F*^2^	Primary atom site location: structure-invariant direct methods
Least-squares matrix: full	Secondary atom site location: difference Fourier map
*R*[*F*^2^ > 2σ(*F*^2^)] = 0.067	Hydrogen site location: inferred from neighbouring sites
*wR*(*F*^2^) = 0.225	H-atom parameters constrained
*S* = 1.00	*w* = 1/[σ^2^(*F*_o_^2^) + (0.1261*P*)^2^] where *P* = (*F*_o_^2^ + 2*F*_c_^2^)/3
15365 reflections	(Δ/σ)_max_ = 0.004
831 parameters	Δρ_max_ = 0.88 e Å^−^^3^
259 restraints	Δρ_min_ = −0.57 e Å^−^^3^

### Special details

**Table:**

Geometry. Bond distances, angles *etc*. have been calculated using the rounded fractional coordinates. All su's are estimated from the variances of the (full) variance-covariance matrix. The cell e.s.d.'s are taken into account in the estimation of distances, angles and torsion angles
Refinement. Refinement of *F*^2^ against ALL reflections. The weighted *R*-factor *wR* and goodness of fit *S* are based on *F*^2^, conventional *R*-factors *R* are based on *F*, with *F* set to zero for negative *F*^2^. The threshold expression of *F*^2^ > σ(*F*^2^) is used only for calculating *R*-factors(gt) *etc*. and is not relevant to the choice of reflections for refinement. *R*-factors based on *F*^2^ are statistically about twice as large as those based on *F*, and *R*- factors based on ALL data will be even larger.

### Fractional atomic coordinates and isotropic or equivalent isotropic displacement parameters (Å^2^)

**Table:**

	*x*	*y*	*z*	*U*_iso_*/*U*_eq_	
Cl1	0.99273 (7)	−0.39783 (6)	0.32740 (4)	0.0719 (3)	
O40	0.52582 (19)	0.46815 (17)	0.31405 (10)	0.0661 (7)	
O43	0.6376 (2)	0.28330 (19)	0.30436 (14)	0.0945 (11)	
O44	0.20936 (17)	0.42683 (15)	0.25533 (10)	0.0611 (6)	
O45	0.39488 (19)	0.04084 (15)	0.27550 (11)	0.0650 (7)	
N2	0.41464 (18)	0.28538 (16)	0.37365 (9)	0.0402 (5)	
N5	0.27486 (19)	0.23366 (17)	0.26915 (10)	0.0444 (5)	
N24	0.33633 (19)	0.03102 (17)	0.45976 (10)	0.0440 (6)	
N25	0.44097 (18)	−0.06358 (17)	0.44880 (9)	0.0416 (6)	
C1	0.4250 (2)	0.3578 (2)	0.32078 (12)	0.0417 (6)	
C3	0.4882 (2)	0.16583 (19)	0.35570 (11)	0.0399 (6)	
C4	0.3811 (2)	0.1361 (2)	0.27651 (12)	0.0430 (6)	
C6	0.2887 (2)	0.3313 (2)	0.26336 (11)	0.0409 (6)	
C7	0.4163 (2)	0.29884 (19)	0.26863 (11)	0.0399 (5)	
C8	0.4720 (2)	0.1727 (2)	0.28676 (11)	0.0397 (5)	
C9	0.4357 (3)	0.3100 (2)	0.43141 (12)	0.0499 (7)	
C10	0.3352 (3)	0.4161 (2)	0.45940 (12)	0.0469 (7)	
C11	0.3575 (3)	0.4766 (3)	0.49836 (15)	0.0679 (10)	
C12	0.2657 (4)	0.5719 (3)	0.52623 (17)	0.0879 (11)	
C13	0.1525 (4)	0.6075 (3)	0.51712 (15)	0.0859 (11)	
C14	0.1278 (3)	0.5487 (3)	0.47838 (15)	0.0740 (10)	
C15	0.2188 (3)	0.4535 (2)	0.44965 (14)	0.0579 (9)	
C16	0.1640 (2)	0.2316 (2)	0.27036 (13)	0.0479 (7)	
C17	0.0737 (3)	0.2876 (3)	0.31495 (15)	0.0740 (13)	
C18	−0.0322 (3)	0.2809 (4)	0.31833 (17)	0.0928 (16)	
C19	−0.0471 (3)	0.2206 (3)	0.27818 (18)	0.0815 (15)	
C20	0.0444 (3)	0.1650 (3)	0.2319 (2)	0.0911 (16)	
C21	0.1498 (3)	0.1710 (3)	0.22826 (17)	0.0713 (11)	
C22	0.4488 (2)	0.0895 (2)	0.39715 (11)	0.0409 (6)	
C23	0.3406 (2)	0.1223 (2)	0.42914 (12)	0.0450 (7)	
C26	0.5105 (2)	−0.0294 (2)	0.41068 (10)	0.0379 (6)	
C27	0.6304 (2)	−0.1164 (2)	0.38973 (11)	0.0385 (6)	
C28	0.6655 (3)	−0.2281 (2)	0.40817 (14)	0.0610 (9)	
C29	0.7764 (3)	−0.3134 (2)	0.39048 (15)	0.0639 (9)	
C30	0.8549 (2)	−0.2896 (2)	0.35297 (12)	0.0472 (7)	
C31	0.8267 (3)	−0.1788 (3)	0.33514 (18)	0.0875 (12)	
C32	0.7165 (3)	−0.0943 (3)	0.35405 (18)	0.0828 (12)	
C33	0.2419 (2)	0.0237 (2)	0.50002 (12)	0.0482 (7)	
C34	0.1347 (3)	0.1166 (3)	0.5099 (2)	0.1044 (13)	
C35	0.0450 (3)	0.1096 (4)	0.5502 (2)	0.1265 (16)	
C36	0.0626 (3)	0.0098 (3)	0.58066 (19)	0.0963 (13)	
C37	0.1694 (3)	−0.0818 (3)	0.57148 (19)	0.0973 (14)	
C38	0.2599 (3)	−0.0766 (3)	0.53084 (16)	0.0732 (10)	
C39	0.5429 (3)	0.3636 (2)	0.31210 (14)	0.0520 (8)	
C41	0.6313 (4)	0.4863 (4)	0.3002 (2)	0.0993 (16)	
C42	0.5998 (4)	0.6009 (4)	0.3140 (2)	0.115 (2)	
Cl1'	0.18611 (12)	0.99456 (13)	−0.21002 (6)	0.1397 (6)	
O40'	0.5436 (2)	0.76797 (18)	0.17379 (12)	0.0793 (10)	
O43'	0.3934 (3)	0.8848 (2)	0.12233 (14)	0.0982 (11)	
O44'	0.52736 (19)	0.44261 (17)	0.17319 (9)	0.0612 (8)	
O45'	0.34709 (17)	0.54348 (15)	−0.00136 (8)	0.0515 (6)	
N2'	0.28545 (19)	0.73543 (18)	0.15662 (9)	0.0438 (6)	
N5'	0.42346 (18)	0.47404 (17)	0.09055 (9)	0.0408 (6)	
N24'	0.0349 (2)	0.66935 (19)	0.08555 (10)	0.0496 (7)	
N25'	0.0304 (2)	0.72321 (19)	0.02929 (10)	0.0512 (8)	
C1'	0.4098 (2)	0.7020 (2)	0.16074 (12)	0.0438 (6)	
C3'	0.2686 (2)	0.7506 (2)	0.09111 (12)	0.0427 (6)	
C4'	0.3813 (2)	0.5558 (2)	0.04336 (11)	0.0401 (7)	
C6'	0.4795 (2)	0.5021 (2)	0.13029 (11)	0.0410 (7)	
C7'	0.4713 (2)	0.6166 (2)	0.10997 (11)	0.0413 (6)	
C8'	0.3883 (2)	0.6590 (2)	0.05994 (11)	0.0410 (6)	
C9'	0.1991 (3)	0.8294 (3)	0.19409 (13)	0.0584 (8)	
C10'	0.2239 (3)	0.8099 (2)	0.26041 (12)	0.0492 (8)	
C11'	0.2617 (3)	0.8750 (3)	0.28592 (14)	0.0611 (10)	
C12'	0.2873 (4)	0.8558 (3)	0.34542 (16)	0.0811 (13)	
C13'	0.2759 (4)	0.7703 (3)	0.38064 (16)	0.0872 (15)	
C14'	0.2399 (4)	0.7033 (3)	0.35620 (16)	0.0884 (13)	
C15'	0.2135 (3)	0.7228 (3)	0.29611 (15)	0.0712 (13)	
C16'	0.4186 (2)	0.3680 (2)	0.09344 (11)	0.0430 (7)	
C17'	0.5183 (3)	0.2670 (2)	0.10030 (13)	0.0536 (8)	
C18'	0.5120 (3)	0.1660 (3)	0.10246 (17)	0.0737 (9)	
C19'	0.4051 (4)	0.1673 (3)	0.09795 (18)	0.0830 (13)	
C20'	0.3065 (3)	0.2682 (3)	0.09114 (19)	0.0809 (13)	
C21'	0.3113 (3)	0.3694 (3)	0.08934 (15)	0.0600 (9)	
C22'	0.1635 (2)	0.7362 (2)	0.08089 (12)	0.0430 (7)	
C23'	0.1146 (2)	0.6758 (2)	0.11719 (12)	0.0460 (8)	
C26'	0.1077 (2)	0.7639 (2)	0.02693 (12)	0.0458 (8)	
C27'	0.1305 (2)	0.8226 (2)	−0.03069 (13)	0.0488 (7)	
C28'	0.1491 (3)	0.7735 (3)	−0.08581 (14)	0.0713 (12)	
C29'	0.1704 (4)	0.8235 (3)	−0.14082 (16)	0.0844 (13)	
C30'	0.1673 (3)	0.9274 (3)	−0.14000 (17)	0.0805 (10)	
C31'	0.1513 (3)	0.9783 (3)	−0.08606 (18)	0.0755 (9)	
C32'	0.1346 (3)	0.9237 (2)	−0.03113 (16)	0.0605 (9)	
C33'	−0.0345 (2)	0.6111 (2)	0.10326 (13)	0.0518 (9)	
C34'	−0.0336 (4)	0.5605 (4)	0.16032 (17)	0.0858 (15)	
C35'	−0.1015 (4)	0.5030 (4)	0.1764 (2)	0.0999 (16)	
C36'	−0.1702 (4)	0.4989 (3)	0.13663 (18)	0.0868 (16)	
C37'	−0.1668 (4)	0.5460 (4)	0.0797 (2)	0.113 (2)	
C38'	−0.0995 (4)	0.6011 (4)	0.06219 (18)	0.0948 (18)	
C39'	0.4460 (3)	0.7962 (3)	0.14954 (14)	0.0559 (9)	
C41'	0.5952 (4)	0.8477 (3)	0.1627 (3)	0.126 (2)	
C42'	0.6759 (7)	0.8153 (7)	0.2110 (4)	0.230 (5)	
H1	0.35830	0.43410	0.32390	0.0500*	
H3	0.57150	0.14300	0.35970	0.0480*	
H7	0.45600	0.31410	0.23000	0.0480*	
H8	0.54770	0.13030	0.26200	0.0480*	
H11	0.43500	0.45340	0.50610	0.0820*	
H12	0.28330	0.61170	0.55180	0.1060*	
H13	0.09190	0.67050	0.53650	0.1030*	
H14	0.04980	0.57260	0.47130	0.0890*	
H15	0.20060	0.41490	0.42360	0.0690*	
H17	0.08310	0.32980	0.34270	0.0890*	
H18	−0.09320	0.31840	0.34870	0.1110*	
H19	−0.11780	0.21600	0.28110	0.0980*	
H20	0.03420	0.12410	0.20370	0.1090*	
H21	0.21060	0.13440	0.19760	0.0860*	
H23	0.27900	0.19590	0.42990	0.0540*	
H28	0.61210	−0.24690	0.43360	0.0730*	
H29	0.79690	−0.38780	0.40450	0.0770*	
H31	0.88180	−0.16120	0.31050	0.1050*	
H32	0.69880	−0.01950	0.34250	0.0990*	
H34	0.12190	0.18520	0.48930	0.1250*	
H35	−0.02790	0.17350	0.55660	0.1520*	
H36	0.00190	0.00460	0.60740	0.1150*	
H37	0.18250	−0.14970	0.59290	0.1170*	
H38	0.33250	−0.14080	0.52450	0.0880*	
H41A	0.69310	0.43300	0.32460	0.1190*	
H41B	0.66120	0.47400	0.25720	0.1190*	
H42D	0.66840	0.61350	0.30520	0.1730*	
H42E	0.57080	0.61230	0.35670	0.1730*	
H42F	0.53920	0.65320	0.28950	0.1730*	
H91	0.50760	0.31860	0.42540	0.0600*	
H92	0.44870	0.24600	0.46010	0.0600*	
H1'	0.43180	0.66330	0.20060	0.0530*	
H9'1	0.12020	0.83850	0.19190	0.0700*	
H9'2	0.20120	0.89890	0.17780	0.0700*	
H3'	0.25720	0.82640	0.07600	0.0510*	
H7'	0.54920	0.61100	0.09430	0.0500*	
H8'	0.41910	0.69170	0.02440	0.0490*	
H11'	0.27020	0.93330	0.26230	0.0730*	
H12'	0.31260	0.90110	0.36190	0.0970*	
H13'	0.29250	0.75770	0.42120	0.1040*	
H14'	0.23310	0.64440	0.38000	0.1060*	
H15'	0.18880	0.67710	0.27970	0.0850*	
H17'	0.59010	0.26640	0.10350	0.0640*	
H18'	0.57950	0.09740	0.10690	0.0880*	
H19'	0.40040	0.09960	0.09950	0.1000*	
H20'	0.23490	0.26860	0.08760	0.0970*	
H21'	0.24340	0.43780	0.08540	0.0720*	
H23'	0.13260	0.64490	0.15630	0.0550*	
H28'	0.14720	0.70470	−0.08580	0.0860*	
H29'	0.18640	0.78780	−0.17740	0.1010*	
H31'	0.15170	1.04790	−0.08640	0.0910*	
H32'	0.12610	0.95600	0.00580	0.0730*	
H34'	0.01190	0.56430	0.18830	0.1030*	
H35'	−0.09960	0.46710	0.21500	0.1200*	
H36'	−0.21870	0.46430	0.14830	0.1040*	
H37'	−0.21140	0.54090	0.05160	0.1350*	
H38'	−0.09770	0.63190	0.02240	0.1140*	
H41C	0.53380	0.92540	0.16650	0.1510*	
H41D	0.63860	0.83960	0.12220	0.1510*	
H42A	0.70100	0.87250	0.21220	0.3450*	
H42B	0.74390	0.74400	0.20190	0.3450*	
H42C	0.63500	0.80850	0.25000	0.3450*	

### Atomic displacement parameters (Å^2^)

**Table:**

	*U*^11^	*U*^22^	*U*^33^	*U*^12^	*U*^13^	*U*^23^
Cl1	0.0458 (5)	0.0517 (4)	0.0859 (6)	0.0009 (3)	0.0072 (4)	−0.0054 (4)
O40	0.0564 (13)	0.0607 (9)	0.0939 (16)	−0.0375 (10)	−0.0080 (11)	−0.0036 (11)
O43	0.0357 (10)	0.0613 (11)	0.171 (3)	−0.0162 (9)	−0.0045 (14)	0.0191 (15)
O44	0.0438 (10)	0.0392 (8)	0.0867 (14)	−0.0064 (8)	−0.0167 (10)	0.0044 (10)
O45	0.0625 (14)	0.0385 (8)	0.0938 (15)	−0.0203 (9)	−0.0183 (12)	−0.0030 (11)
N2	0.0364 (11)	0.0327 (8)	0.0446 (8)	−0.0112 (8)	−0.0053 (8)	0.0045 (6)
N5	0.0361 (8)	0.0405 (7)	0.0519 (12)	−0.0138 (7)	−0.0048 (9)	−0.0011 (10)
N24	0.0363 (9)	0.0383 (9)	0.0452 (12)	−0.0097 (7)	−0.0001 (8)	0.0059 (8)
N25	0.0366 (9)	0.0355 (8)	0.0427 (12)	−0.0105 (7)	−0.0010 (8)	0.0053 (8)
C1	0.0355 (10)	0.0324 (9)	0.0530 (10)	−0.0138 (9)	−0.0041 (10)	0.0060 (8)
C3	0.0293 (12)	0.0318 (8)	0.0488 (10)	−0.0075 (9)	−0.0038 (8)	0.0061 (6)
C4	0.0388 (10)	0.0360 (8)	0.0485 (13)	−0.0141 (8)	−0.0014 (11)	0.0013 (12)
C6	0.0342 (10)	0.0369 (8)	0.0431 (13)	−0.0104 (7)	−0.0030 (10)	0.0029 (11)
C7	0.0341 (9)	0.0348 (9)	0.0431 (9)	−0.0127 (8)	0.0014 (10)	0.0066 (8)
C8	0.0303 (9)	0.0327 (8)	0.0457 (8)	−0.0085 (7)	0.0029 (10)	0.0020 (8)
C9	0.0500 (13)	0.0464 (13)	0.0524 (11)	−0.0207 (10)	−0.0108 (12)	0.0019 (9)
C10	0.0576 (11)	0.0405 (12)	0.0418 (13)	−0.0231 (10)	−0.0053 (11)	0.0044 (8)
C11	0.0867 (18)	0.0617 (17)	0.0631 (19)	−0.0383 (14)	−0.0120 (15)	−0.0079 (12)
C12	0.127 (2)	0.0618 (19)	0.069 (2)	−0.0348 (18)	−0.012 (2)	−0.0181 (14)
C13	0.1104 (19)	0.0545 (19)	0.0526 (19)	−0.0069 (18)	0.0003 (18)	−0.0021 (13)
C14	0.0654 (15)	0.0603 (18)	0.066 (2)	−0.0073 (12)	0.0003 (15)	0.0053 (13)
C15	0.0548 (12)	0.0467 (15)	0.0644 (18)	−0.0176 (11)	−0.0078 (13)	0.0029 (11)
C16	0.0397 (9)	0.0518 (15)	0.0510 (14)	−0.0206 (10)	−0.0068 (9)	0.0030 (10)
C17	0.0485 (15)	0.114 (3)	0.0647 (19)	−0.0408 (18)	0.0053 (12)	−0.0236 (16)
C18	0.0514 (17)	0.164 (4)	0.071 (2)	−0.056 (2)	0.0044 (14)	−0.0201 (19)
C19	0.0572 (18)	0.106 (3)	0.097 (3)	−0.051 (2)	−0.0158 (14)	0.0085 (17)
C20	0.059 (2)	0.095 (3)	0.130 (3)	−0.036 (2)	−0.0201 (16)	−0.032 (2)
C21	0.0515 (16)	0.078 (2)	0.088 (2)	−0.0281 (17)	−0.0078 (14)	−0.0256 (16)
C22	0.0357 (11)	0.0341 (8)	0.0440 (12)	−0.0103 (8)	−0.0026 (9)	0.0047 (9)
C23	0.0376 (11)	0.0357 (9)	0.0490 (15)	−0.0092 (8)	0.0010 (10)	0.0052 (10)
C26	0.0377 (9)	0.0353 (8)	0.0317 (12)	−0.0108 (7)	−0.0029 (8)	0.0053 (9)
C27	0.0378 (10)	0.0342 (8)	0.0345 (12)	−0.0103 (7)	−0.0022 (9)	0.0020 (9)
C28	0.0506 (14)	0.0372 (9)	0.073 (2)	−0.0106 (9)	0.0170 (13)	0.0092 (13)
C29	0.0538 (14)	0.0348 (11)	0.077 (2)	−0.0060 (8)	0.0112 (13)	0.0088 (13)
C30	0.0372 (11)	0.0403 (9)	0.0478 (15)	−0.0053 (8)	−0.0025 (9)	0.0003 (11)
C31	0.0523 (16)	0.0482 (10)	0.117 (3)	−0.0026 (10)	0.0322 (17)	0.0227 (18)
C32	0.0513 (16)	0.0400 (11)	0.116 (3)	−0.0030 (9)	0.0279 (16)	0.0257 (17)
C33	0.0376 (11)	0.0519 (12)	0.0438 (15)	−0.0144 (9)	0.0010 (9)	0.0068 (11)
C34	0.0508 (17)	0.0717 (18)	0.129 (3)	0.0025 (12)	0.0331 (19)	0.039 (2)
C35	0.053 (2)	0.102 (2)	0.150 (4)	0.0010 (17)	0.041 (2)	0.048 (3)
C36	0.0572 (18)	0.102 (2)	0.104 (3)	−0.0301 (15)	0.0238 (19)	0.024 (2)
C37	0.067 (2)	0.084 (2)	0.114 (3)	−0.0288 (14)	0.022 (2)	0.034 (2)
C38	0.0505 (16)	0.0581 (14)	0.090 (2)	−0.0177 (12)	0.0116 (14)	0.0205 (15)
C39	0.0395 (11)	0.0479 (10)	0.0669 (18)	−0.0207 (8)	−0.0076 (12)	0.0098 (13)
C41	0.079 (2)	0.108 (2)	0.146 (4)	−0.071 (2)	−0.020 (2)	−0.001 (3)
C42	0.137 (4)	0.121 (3)	0.145 (4)	−0.104 (3)	−0.034 (3)	0.004 (3)
Cl1'	0.1165 (10)	0.1634 (12)	0.1110 (9)	−0.0587 (9)	−0.0035 (7)	0.0712 (9)
O40'	0.0692 (15)	0.0680 (14)	0.125 (2)	−0.0462 (12)	−0.0362 (13)	0.0044 (13)
O43'	0.106 (2)	0.0794 (15)	0.141 (2)	−0.0654 (16)	−0.0595 (17)	0.0412 (15)
O44'	0.0796 (16)	0.0643 (12)	0.0524 (11)	−0.0411 (12)	−0.0272 (10)	0.0127 (9)
O45'	0.0554 (12)	0.0591 (11)	0.0388 (9)	−0.0232 (10)	−0.0085 (8)	−0.0077 (8)
N2'	0.0392 (9)	0.0510 (12)	0.0437 (8)	−0.0217 (9)	−0.0044 (7)	−0.0079 (8)
N5'	0.0392 (12)	0.0475 (8)	0.0379 (10)	−0.0222 (9)	−0.0025 (8)	−0.0015 (8)
N24'	0.0433 (13)	0.0610 (14)	0.0512 (11)	−0.0288 (11)	−0.0075 (9)	−0.0022 (9)
N25'	0.0463 (14)	0.0624 (14)	0.0525 (11)	−0.0300 (11)	−0.0132 (9)	0.0017 (10)
C1'	0.0426 (10)	0.0542 (11)	0.0429 (11)	−0.0283 (9)	−0.0082 (9)	−0.0014 (9)
C3'	0.0396 (10)	0.0444 (12)	0.0464 (9)	−0.0200 (8)	−0.0096 (8)	−0.0005 (9)
C4'	0.0365 (14)	0.0501 (10)	0.0338 (11)	−0.0207 (11)	−0.0004 (8)	−0.0030 (7)
C6'	0.0381 (15)	0.0497 (10)	0.0384 (12)	−0.0235 (11)	−0.0038 (9)	0.0007 (8)
C7'	0.0340 (10)	0.0508 (10)	0.0439 (12)	−0.0241 (8)	−0.0034 (7)	0.0004 (7)
C8'	0.0394 (10)	0.0501 (10)	0.0369 (9)	−0.0247 (9)	−0.0027 (7)	0.0021 (7)
C9'	0.0509 (14)	0.0591 (16)	0.0616 (10)	−0.0195 (12)	−0.0037 (11)	−0.0192 (9)
C10'	0.0483 (17)	0.0491 (15)	0.0522 (9)	−0.0247 (13)	0.0071 (11)	−0.0166 (9)
C11'	0.085 (2)	0.0578 (18)	0.0555 (13)	−0.0450 (17)	−0.0045 (15)	−0.0081 (12)
C12'	0.111 (3)	0.088 (2)	0.0618 (16)	−0.056 (2)	−0.0160 (19)	−0.0147 (15)
C13'	0.111 (3)	0.094 (3)	0.0454 (16)	−0.040 (2)	0.0015 (17)	−0.0057 (14)
C14'	0.123 (3)	0.072 (2)	0.0636 (15)	−0.049 (2)	0.0254 (18)	−0.0033 (15)
C15'	0.093 (3)	0.0675 (19)	0.0682 (14)	−0.055 (2)	0.0236 (16)	−0.0202 (13)
C16'	0.0453 (12)	0.0479 (9)	0.0381 (13)	−0.0239 (9)	−0.0005 (11)	−0.0049 (10)
C17'	0.0466 (12)	0.0501 (9)	0.0608 (17)	−0.0215 (9)	−0.0002 (13)	−0.0003 (14)
C18'	0.0722 (17)	0.0481 (10)	0.096 (2)	−0.0252 (13)	−0.006 (2)	−0.0002 (17)
C19'	0.093 (2)	0.0553 (12)	0.116 (3)	−0.0464 (14)	−0.015 (2)	−0.001 (2)
C20'	0.0733 (18)	0.0692 (15)	0.120 (3)	−0.0486 (13)	−0.017 (2)	0.002 (2)
C21'	0.0487 (13)	0.0548 (12)	0.083 (2)	−0.0282 (11)	−0.0091 (16)	−0.0048 (16)
C22'	0.0373 (11)	0.0462 (14)	0.0465 (11)	−0.0184 (10)	−0.0051 (9)	−0.0087 (10)
C23'	0.0392 (15)	0.0540 (16)	0.0466 (11)	−0.0220 (12)	−0.0059 (10)	−0.0050 (11)
C26'	0.0381 (15)	0.0487 (14)	0.0520 (10)	−0.0196 (12)	−0.0106 (9)	−0.0011 (10)
C27'	0.0376 (15)	0.0534 (14)	0.0558 (9)	−0.0202 (13)	−0.0144 (11)	0.0057 (9)
C28'	0.092 (3)	0.088 (2)	0.0516 (10)	−0.054 (2)	−0.0213 (17)	0.0060 (11)
C29'	0.091 (3)	0.108 (2)	0.0539 (11)	−0.046 (2)	−0.0200 (18)	0.0158 (13)
C30'	0.051 (2)	0.095 (2)	0.0797 (11)	−0.027 (2)	−0.0111 (18)	0.0342 (13)
C31'	0.0502 (19)	0.0660 (18)	0.1050 (11)	−0.0282 (17)	−0.0058 (19)	0.0224 (13)
C32'	0.0459 (17)	0.0531 (15)	0.0804 (12)	−0.0226 (14)	−0.0057 (15)	0.0036 (11)
C33'	0.0412 (16)	0.0592 (17)	0.0589 (13)	−0.0271 (13)	0.0006 (11)	−0.0073 (12)
C34'	0.096 (3)	0.123 (3)	0.0724 (17)	−0.081 (2)	−0.0190 (19)	0.0219 (19)
C35'	0.112 (3)	0.122 (3)	0.099 (2)	−0.087 (3)	−0.016 (2)	0.029 (2)
C36'	0.098 (3)	0.098 (3)	0.096 (2)	−0.076 (3)	0.0174 (19)	−0.020 (2)
C37'	0.142 (4)	0.181 (5)	0.090 (2)	−0.137 (4)	−0.010 (2)	−0.007 (3)
C38'	0.116 (3)	0.156 (4)	0.0716 (19)	−0.112 (3)	−0.020 (2)	0.010 (2)
C39'	0.0579 (17)	0.0580 (12)	0.0654 (19)	−0.0362 (14)	−0.0133 (13)	−0.0021 (11)
C41'	0.108 (3)	0.079 (3)	0.236 (6)	−0.070 (3)	−0.060 (4)	−0.004 (3)
C42'	0.239 (8)	0.261 (8)	0.319 (10)	−0.196 (8)	−0.152 (7)	0.025 (7)

### Geometric parameters (Å, °)

**Table:**

Cl1—C30	1.741 (3)	C21—H21	0.9300
Cl1'—C30'	1.746 (4)	C23—H23	0.9300
O40—C39	1.312 (3)	C28—H28	0.9300
O40—C41	1.467 (6)	C29—H29	0.9300
O43—C39	1.191 (4)	C31—H31	0.9300
O44—C6	1.220 (3)	C32—H32	0.9300
O45—C4	1.202 (3)	C34—H34	0.9300
O40'—C41'	1.478 (6)	C35—H35	0.9300
O40'—C39'	1.308 (5)	C36—H36	0.9300
O43'—C39'	1.199 (4)	C37—H37	0.9300
O44'—C6'	1.208 (3)	C38—H38	0.9300
O45'—C4'	1.193 (3)	C41—H41A	0.9700
N2—C1	1.466 (3)	C41—H41B	0.9700
N2—C3	1.488 (3)	C42—H42E	0.9600
N2—C9	1.442 (4)	C42—H42F	0.9600
N5—C16	1.432 (4)	C42—H42D	0.9600
N5—C6	1.386 (3)	C1'—C7'	1.534 (3)
N5—C4	1.395 (3)	C1'—C39'	1.517 (5)
N24—N25	1.358 (3)	C3'—C8'	1.564 (4)
N24—C23	1.349 (3)	C3'—C22'	1.499 (4)
N24—C33	1.430 (4)	C4'—C8'	1.507 (4)
N25—C26	1.338 (3)	C6'—C7'	1.505 (3)
N2'—C1'	1.457 (4)	C7'—C8'	1.524 (4)
N2'—C3'	1.472 (3)	C9'—C10'	1.513 (4)
N2'—C9'	1.463 (4)	C10'—C11'	1.373 (5)
N5'—C16'	1.440 (3)	C10'—C15'	1.382 (5)
N5'—C4'	1.393 (3)	C11'—C12'	1.369 (5)
N5'—C6'	1.390 (4)	C12'—C13'	1.365 (6)
N24'—C23'	1.358 (4)	C13'—C14'	1.366 (7)
N24'—C33'	1.424 (4)	C14'—C15'	1.385 (5)
N24'—N25'	1.362 (3)	C16'—C17'	1.372 (4)
N25'—C26'	1.328 (4)	C16'—C21'	1.384 (5)
C1—C7	1.506 (4)	C17'—C18'	1.383 (5)
C1—C39	1.534 (5)	C18'—C19'	1.382 (7)
C3—C8	1.553 (3)	C19'—C20'	1.364 (6)
C3—C22	1.509 (4)	C20'—C21'	1.377 (5)
C4—C8	1.511 (4)	C22'—C23'	1.368 (4)
C6—C7	1.505 (4)	C22'—C26'	1.406 (4)
C7—C8	1.524 (3)	C26'—C27'	1.487 (4)
C9—C10	1.517 (4)	C27'—C28'	1.381 (4)
C10—C15	1.379 (6)	C27'—C32'	1.374 (4)
C10—C11	1.382 (5)	C28'—C29'	1.377 (5)
C11—C12	1.393 (5)	C29'—C30'	1.371 (5)
C12—C13	1.341 (7)	C30'—C31'	1.376 (5)
C13—C14	1.379 (6)	C31'—C32'	1.389 (5)
C14—C15	1.397 (5)	C33'—C34'	1.363 (5)
C16—C17	1.373 (5)	C33'—C38'	1.373 (6)
C16—C21	1.378 (5)	C34'—C35'	1.396 (8)
C17—C18	1.393 (6)	C35'—C36'	1.354 (7)
C18—C19	1.347 (6)	C36'—C37'	1.348 (6)
C19—C20	1.404 (6)	C37'—C38'	1.367 (8)
C20—C21	1.382 (6)	C41'—C42'	1.484 (11)
C22—C26	1.425 (3)	C1'—H1'	0.9800
C22—C23	1.353 (4)	C3'—H3'	0.9800
C26—C27	1.473 (4)	C7'—H7'	0.9800
C27—C32	1.388 (5)	C8'—H8'	0.9800
C27—C28	1.375 (3)	C9'—H9'1	0.9700
C28—C29	1.376 (4)	C9'—H9'2	0.9700
C29—C30	1.341 (5)	C11'—H11'	0.9300
C30—C31	1.375 (4)	C12'—H12'	0.9300
C31—C32	1.373 (5)	C13'—H13'	0.9300
C33—C38	1.368 (4)	C14'—H14'	0.9300
C33—C34	1.362 (5)	C15'—H15'	0.9300
C34—C35	1.381 (6)	C17'—H17'	0.9300
C35—C36	1.361 (6)	C18'—H18'	0.9300
C36—C37	1.350 (5)	C19'—H19'	0.9300
C37—C38	1.384 (6)	C20'—H20'	0.9300
C41—C42	1.443 (7)	C21'—H21'	0.9300
C1—H1	0.9800	C23'—H23'	0.9300
C3—H3	0.9800	C28'—H28'	0.9300
C7—H7	0.9800	C29'—H29'	0.9300
C8—H8	0.9800	C31'—H31'	0.9300
C9—H91	0.9700	C32'—H32'	0.9300
C9—H92	0.9700	C34'—H34'	0.9300
C11—H11	0.9300	C35'—H35'	0.9300
C12—H12	0.9300	C36'—H36'	0.9300
C13—H13	0.9300	C37'—H37'	0.9300
C14—H14	0.9300	C38'—H38'	0.9300
C15—H15	0.9300	C41'—H41C	0.9700
C17—H17	0.9300	C41'—H41D	0.9700
C18—H18	0.9300	C42'—H42A	0.9600
C19—H19	0.9300	C42'—H42B	0.9600
C20—H20	0.9300	C42'—H42C	0.9600
			
C39—O40—C41	116.5 (3)	O40—C41—H41A	110.00
C39'—O40'—C41'	117.1 (3)	O40—C41—H41B	110.00
C1—N2—C9	115.9 (2)	C42—C41—H41A	110.00
C3—N2—C9	114.8 (2)	C42—C41—H41B	110.00
C1—N2—C3	107.05 (18)	H41A—C41—H41B	108.00
C4—N5—C6	112.4 (2)	C41—C42—H42F	110.00
C4—N5—C16	122.9 (2)	H42E—C42—H42F	109.00
C6—N5—C16	124.6 (2)	H42D—C42—H42E	109.00
N25—N24—C23	110.7 (2)	H42D—C42—H42F	110.00
N25—N24—C33	119.8 (2)	C41—C42—H42D	110.00
C23—N24—C33	129.5 (2)	C41—C42—H42E	109.00
N24—N25—C26	105.6 (2)	N2'—C1'—C7'	101.5 (2)
C1'—N2'—C9'	116.1 (2)	N2'—C1'—C39'	116.0 (2)
C3'—N2'—C9'	114.7 (2)	C7'—C1'—C39'	110.1 (2)
C1'—N2'—C3'	107.4 (2)	N2'—C3'—C8'	103.9 (2)
C6'—N5'—C16'	124.6 (2)	N2'—C3'—C22'	110.9 (2)
C4'—N5'—C6'	112.5 (2)	C8'—C3'—C22'	113.1 (2)
C4'—N5'—C16'	122.7 (2)	O45'—C4'—N5'	125.0 (2)
C23'—N24'—C33'	128.2 (2)	O45'—C4'—C8'	127.9 (2)
N25'—N24'—C23'	111.4 (2)	N5'—C4'—C8'	107.1 (2)
N25'—N24'—C33'	120.3 (2)	O44'—C6'—N5'	124.8 (2)
N24'—N25'—C26'	104.7 (2)	O44'—C6'—C7'	126.8 (3)
N2—C1—C39	113.5 (2)	N5'—C6'—C7'	108.4 (2)
C7—C1—C39	110.6 (2)	C1'—C7'—C6'	112.0 (2)
N2—C1—C7	101.6 (2)	C1'—C7'—C8'	105.9 (2)
N2—C3—C8	102.79 (18)	C6'—C7'—C8'	104.3 (2)
N2—C3—C22	109.7 (2)	C3'—C8'—C4'	113.5 (2)
C8—C3—C22	116.5 (2)	C3'—C8'—C7'	104.4 (2)
O45—C4—N5	124.9 (3)	C4'—C8'—C7'	104.93 (19)
O45—C4—C8	127.3 (3)	N2'—C9'—C10'	111.9 (3)
N5—C4—C8	107.8 (2)	C9'—C10'—C11'	121.1 (3)
O44—C6—N5	124.7 (3)	C9'—C10'—C15'	120.6 (3)
O44—C6—C7	126.7 (2)	C11'—C10'—C15'	118.3 (3)
N5—C6—C7	108.6 (2)	C10'—C11'—C12'	121.3 (3)
C6—C7—C8	104.8 (2)	C11'—C12'—C13'	120.1 (4)
C1—C7—C8	106.6 (2)	C12'—C13'—C14'	119.8 (4)
C1—C7—C6	110.3 (2)	C13'—C14'—C15'	120.2 (4)
C3—C8—C4	112.1 (2)	C10'—C15'—C14'	120.2 (4)
C3—C8—C7	105.01 (19)	N5'—C16'—C17'	120.8 (3)
C4—C8—C7	104.6 (2)	N5'—C16'—C21'	118.9 (2)
N2—C9—C10	113.8 (3)	C17'—C16'—C21'	120.3 (3)
C9—C10—C15	122.4 (3)	C16'—C17'—C18'	119.9 (4)
C11—C10—C15	117.3 (3)	C17'—C18'—C19'	119.8 (3)
C9—C10—C11	120.3 (3)	C18'—C19'—C20'	119.8 (4)
C10—C11—C12	121.0 (4)	C19'—C20'—C21'	121.0 (4)
C11—C12—C13	121.5 (4)	C16'—C21'—C20'	119.2 (3)
C12—C13—C14	118.6 (4)	C3'—C22'—C23'	127.5 (2)
C13—C14—C15	120.7 (4)	C3'—C22'—C26'	127.0 (2)
C10—C15—C14	120.9 (3)	C23'—C22'—C26'	104.6 (2)
N5—C16—C17	119.3 (3)	N24'—C23'—C22'	107.5 (2)
N5—C16—C21	120.1 (3)	N25'—C26'—C22'	111.9 (2)
C17—C16—C21	120.6 (3)	N25'—C26'—C27'	118.9 (2)
C16—C17—C18	119.6 (3)	C22'—C26'—C27'	129.1 (3)
C17—C18—C19	120.7 (4)	C26'—C27'—C28'	118.9 (2)
C18—C19—C20	119.7 (4)	C26'—C27'—C32'	122.4 (3)
C19—C20—C21	119.9 (4)	C28'—C27'—C32'	118.7 (3)
C16—C21—C20	119.5 (3)	C27'—C28'—C29'	121.9 (3)
C3—C22—C26	130.1 (2)	C28'—C29'—C30'	118.0 (3)
C23—C22—C26	104.4 (2)	Cl1'—C30'—C29'	118.1 (3)
C3—C22—C23	125.5 (2)	Cl1'—C30'—C31'	120.0 (3)
N24—C23—C22	109.0 (2)	C29'—C30'—C31'	121.9 (3)
N25—C26—C27	117.4 (2)	C30'—C31'—C32'	118.7 (3)
C22—C26—C27	132.2 (2)	C27'—C32'—C31'	120.7 (3)
N25—C26—C22	110.3 (2)	N24'—C33'—C34'	120.6 (3)
C28—C27—C32	115.4 (3)	N24'—C33'—C38'	120.4 (3)
C26—C27—C28	119.8 (3)	C34'—C33'—C38'	119.0 (4)
C26—C27—C32	124.7 (2)	C33'—C34'—C35'	119.5 (4)
C27—C28—C29	122.7 (3)	C34'—C35'—C36'	120.9 (4)
C28—C29—C30	120.2 (2)	C35'—C36'—C37'	118.7 (5)
Cl1—C30—C31	119.8 (3)	C36'—C37'—C38'	121.7 (5)
C29—C30—C31	119.7 (3)	C33'—C38'—C37'	120.0 (4)
Cl1—C30—C29	120.45 (19)	O40'—C39'—O43'	123.9 (4)
C30—C31—C32	119.4 (4)	O40'—C39'—C1'	110.7 (3)
C27—C32—C31	122.4 (3)	O43'—C39'—C1'	125.4 (4)
N24—C33—C34	120.8 (3)	O40'—C41'—C42'	104.3 (5)
N24—C33—C38	120.0 (3)	N2'—C1'—H1'	110.00
C34—C33—C38	119.2 (3)	C7'—C1'—H1'	110.00
C33—C34—C35	120.5 (4)	C39'—C1'—H1'	110.00
C34—C35—C36	120.4 (4)	N2'—C3'—H3'	110.00
C35—C36—C37	119.0 (4)	C8'—C3'—H3'	110.00
C36—C37—C38	121.3 (4)	C22'—C3'—H3'	110.00
C33—C38—C37	119.6 (3)	C1'—C7'—H7'	111.00
O40—C39—O43	124.6 (4)	C6'—C7'—H7'	111.00
O40—C39—C1	111.3 (3)	C8'—C7'—H7'	111.00
O43—C39—C1	124.1 (3)	C3'—C8'—H8'	111.00
O40—C41—C42	108.9 (4)	C4'—C8'—H8'	111.00
C7—C1—H1	110.00	C7'—C8'—H8'	111.00
C39—C1—H1	110.00	N2'—C9'—H9'1	109.00
N2—C1—H1	110.00	N2'—C9'—H9'2	109.00
N2—C3—H3	109.00	C10'—C9'—H9'1	109.00
C8—C3—H3	109.00	C10'—C9'—H9'2	109.00
C22—C3—H3	109.00	H9'1—C9'—H9'2	108.00
C6—C7—H7	112.00	C10'—C11'—H11'	119.00
C8—C7—H7	112.00	C12'—C11'—H11'	119.00
C1—C7—H7	112.00	C11'—C12'—H12'	120.00
C4—C8—H8	112.00	C13'—C12'—H12'	120.00
C7—C8—H8	112.00	C12'—C13'—H13'	120.00
C3—C8—H8	112.00	C14'—C13'—H13'	120.00
H91—C9—H92	108.00	C13'—C14'—H14'	120.00
C10—C9—H92	109.00	C15'—C14'—H14'	120.00
C10—C9—H91	109.00	C10'—C15'—H15'	120.00
N2—C9—H91	109.00	C14'—C15'—H15'	120.00
N2—C9—H92	109.00	C16'—C17'—H17'	120.00
C12—C11—H11	119.00	C18'—C17'—H17'	120.00
C10—C11—H11	120.00	C17'—C18'—H18'	120.00
C13—C12—H12	119.00	C19'—C18'—H18'	120.00
C11—C12—H12	119.00	C18'—C19'—H19'	120.00
C12—C13—H13	121.00	C20'—C19'—H19'	120.00
C14—C13—H13	121.00	C19'—C20'—H20'	119.00
C15—C14—H14	120.00	C21'—C20'—H20'	120.00
C13—C14—H14	120.00	C16'—C21'—H21'	120.00
C10—C15—H15	120.00	C20'—C21'—H21'	120.00
C14—C15—H15	120.00	N24'—C23'—H23'	126.00
C16—C17—H17	120.00	C22'—C23'—H23'	126.00
C18—C17—H17	120.00	C27'—C28'—H28'	119.00
C17—C18—H18	120.00	C29'—C28'—H28'	119.00
C19—C18—H18	120.00	C28'—C29'—H29'	121.00
C20—C19—H19	120.00	C30'—C29'—H29'	121.00
C18—C19—H19	120.00	C30'—C31'—H31'	121.00
C21—C20—H20	120.00	C32'—C31'—H31'	121.00
C19—C20—H20	120.00	C27'—C32'—H32'	120.00
C16—C21—H21	120.00	C31'—C32'—H32'	120.00
C20—C21—H21	120.00	C33'—C34'—H34'	120.00
C22—C23—H23	126.00	C35'—C34'—H34'	120.00
N24—C23—H23	125.00	C34'—C35'—H35'	120.00
C29—C28—H28	119.00	C36'—C35'—H35'	120.00
C27—C28—H28	119.00	C35'—C36'—H36'	121.00
C28—C29—H29	120.00	C37'—C36'—H36'	121.00
C30—C29—H29	120.00	C36'—C37'—H37'	119.00
C30—C31—H31	120.00	C38'—C37'—H37'	119.00
C32—C31—H31	120.00	C33'—C38'—H38'	120.00
C31—C32—H32	119.00	C37'—C38'—H38'	120.00
C27—C32—H32	119.00	O40'—C41'—H41C	111.00
C33—C34—H34	120.00	O40'—C41'—H41D	111.00
C35—C34—H34	120.00	C42'—C41'—H41C	111.00
C36—C35—H35	120.00	C42'—C41'—H41D	111.00
C34—C35—H35	120.00	H41C—C41'—H41D	109.00
C35—C36—H36	121.00	C41'—C42'—H42A	109.00
C37—C36—H36	120.00	C41'—C42'—H42B	109.00
C36—C37—H37	119.00	C41'—C42'—H42C	109.00
C38—C37—H37	119.00	H42A—C42'—H42B	110.00
C37—C38—H38	120.00	H42A—C42'—H42C	109.00
C33—C38—H38	120.00	H42B—C42'—H42C	110.00
			
C41—O40—C39—O43	5.6 (5)	C21—C16—C17—C18	1.4 (5)
C41—O40—C39—C1	−174.1 (3)	N5—C16—C21—C20	176.7 (3)
C39—O40—C41—C42	−170.8 (3)	C17—C16—C21—C20	−1.3 (5)
C39'—O40'—C41'—C42'	161.3 (4)	C16—C17—C18—C19	−0.3 (6)
C41'—O40'—C39'—O43'	−4.5 (5)	C17—C18—C19—C20	−0.8 (6)
C41'—O40'—C39'—C1'	175.5 (3)	C18—C19—C20—C21	0.9 (6)
C3—N2—C1—C39	−76.4 (3)	C19—C20—C21—C16	0.1 (5)
C1—N2—C3—C8	−35.2 (3)	C3—C22—C26—N25	−178.3 (2)
C9—N2—C1—C7	171.9 (2)	C23—C22—C26—C27	178.4 (3)
C9—N2—C1—C39	53.2 (3)	C3—C22—C26—C27	0.2 (5)
C3—N2—C1—C7	42.3 (3)	C23—C22—C26—N25	0.0 (3)
C1—N2—C9—C10	74.3 (3)	C26—C22—C23—N24	0.0 (3)
C3—N2—C9—C10	−160.0 (2)	C3—C22—C23—N24	178.4 (2)
C9—N2—C3—C8	−165.4 (2)	N25—C26—C27—C28	1.7 (4)
C9—N2—C3—C22	70.1 (3)	N25—C26—C27—C32	−174.9 (3)
C1—N2—C3—C22	−159.7 (2)	C22—C26—C27—C28	−176.6 (3)
C6—N5—C4—O45	−171.1 (3)	C22—C26—C27—C32	6.8 (5)
C6—N5—C4—C8	9.8 (3)	C32—C27—C28—C29	−2.8 (5)
C4—N5—C6—C7	−1.4 (3)	C26—C27—C32—C31	−179.3 (3)
C16—N5—C6—O44	−4.1 (4)	C26—C27—C28—C29	−179.7 (3)
C16—N5—C6—C7	175.9 (2)	C28—C27—C32—C31	4.0 (5)
C4—N5—C16—C17	118.1 (3)	C27—C28—C29—C30	−1.1 (5)
C4—N5—C16—C21	−60.0 (4)	C28—C29—C30—C31	3.8 (5)
C6—N5—C16—C17	−59.0 (4)	C28—C29—C30—Cl1	−176.9 (3)
C6—N5—C16—C21	123.0 (3)	C29—C30—C31—C32	−2.6 (5)
C16—N5—C4—C8	−167.6 (2)	Cl1—C30—C31—C32	178.1 (3)
C4—N5—C6—O44	178.6 (2)	C30—C31—C32—C27	−1.4 (6)
C16—N5—C4—O45	11.5 (4)	N24—C33—C34—C35	−178.1 (3)
N25—N24—C23—C22	0.0 (3)	C38—C33—C34—C35	−0.4 (6)
N25—N24—C33—C34	−177.5 (3)	N24—C33—C38—C37	177.6 (3)
N25—N24—C33—C38	4.8 (4)	C34—C33—C38—C37	−0.2 (5)
C33—N24—C23—C22	179.1 (3)	C33—C34—C35—C36	0.1 (7)
C23—N24—N25—C26	−0.1 (3)	C34—C35—C36—C37	0.8 (7)
C33—N24—N25—C26	−179.2 (2)	C35—C36—C37—C38	−1.4 (6)
C23—N24—C33—C38	−174.2 (3)	C36—C37—C38—C33	1.2 (6)
C23—N24—C33—C34	3.5 (5)	N2'—C1'—C7'—C6'	−79.5 (3)
N24—N25—C26—C22	0.1 (3)	N2'—C1'—C7'—C8'	33.6 (2)
N24—N25—C26—C27	−178.7 (2)	C39'—C1'—C7'—C6'	157.1 (3)
C3'—N2'—C1'—C39'	77.0 (3)	C39'—C1'—C7'—C8'	−89.8 (3)
C9'—N2'—C1'—C7'	−172.1 (2)	N2'—C1'—C39'—O40'	158.6 (2)
C9'—N2'—C1'—C39'	−52.9 (3)	N2'—C1'—C39'—O43'	−21.4 (4)
C1'—N2'—C3'—C8'	34.1 (2)	C7'—C1'—C39'—O40'	−87.0 (3)
C1'—N2'—C9'—C10'	−51.9 (4)	C7'—C1'—C39'—O43'	93.0 (4)
C3'—N2'—C9'—C10'	−178.2 (3)	N2'—C3'—C8'—C4'	102.3 (2)
C9'—N2'—C3'—C8'	164.7 (3)	N2'—C3'—C8'—C7'	−11.3 (3)
C9'—N2'—C3'—C22'	−73.4 (3)	C22'—C3'—C8'—C4'	−18.1 (3)
C1'—N2'—C3'—C22'	155.9 (2)	C22'—C3'—C8'—C7'	−131.7 (2)
C3'—N2'—C1'—C7'	−42.3 (2)	N2'—C3'—C22'—C23'	−24.9 (3)
C6'—N5'—C4'—O45'	167.6 (3)	N2'—C3'—C22'—C26'	167.8 (2)
C6'—N5'—C4'—C8'	−12.1 (3)	C8'—C3'—C22'—C23'	91.4 (3)
C16'—N5'—C6'—C7'	176.6 (2)	C8'—C3'—C22'—C26'	−75.9 (3)
C4'—N5'—C16'—C17'	127.5 (3)	O45'—C4'—C8'—C3'	83.4 (3)
C4'—N5'—C16'—C21'	−52.7 (4)	O45'—C4'—C8'—C7'	−163.2 (3)
C6'—N5'—C16'—C17'	−46.2 (4)	N5'—C4'—C8'—C3'	−96.8 (3)
C6'—N5'—C16'—C21'	133.6 (3)	N5'—C4'—C8'—C7'	16.6 (3)
C16'—N5'—C4'—C8'	173.5 (2)	O44'—C6'—C7'—C1'	−58.5 (4)
C4'—N5'—C6'—O44'	−176.9 (3)	O44'—C6'—C7'—C8'	−172.6 (3)
C4'—N5'—C6'—C7'	2.3 (3)	N5'—C6'—C7'—C1'	122.3 (2)
C16'—N5'—C6'—O44'	−2.6 (4)	N5'—C6'—C7'—C8'	8.2 (3)
C16'—N5'—C4'—O45'	−6.8 (4)	C1'—C7'—C8'—C3'	−13.5 (3)
N25'—N24'—C33'—C34'	−177.6 (3)	C1'—C7'—C8'—C4'	−133.1 (2)
N25'—N24'—C33'—C38'	5.1 (4)	C6'—C7'—C8'—C3'	104.9 (2)
C23'—N24'—N25'—C26'	−0.6 (3)	C6'—C7'—C8'—C4'	−14.8 (3)
C33'—N24'—N25'—C26'	−177.6 (2)	N2'—C9'—C10'—C11'	111.5 (4)
N25'—N24'—C23'—C22'	0.4 (3)	N2'—C9'—C10'—C15'	−65.9 (5)
C33'—N24'—C23'—C22'	177.1 (2)	C9'—C10'—C11'—C12'	−178.2 (4)
C23'—N24'—C33'—C34'	5.9 (5)	C15'—C10'—C11'—C12'	−0.8 (6)
C23'—N24'—C33'—C38'	−171.4 (3)	C9'—C10'—C15'—C14'	178.0 (4)
N24'—N25'—C26'—C27'	176.4 (2)	C11'—C10'—C15'—C14'	0.5 (6)
N24'—N25'—C26'—C22'	0.6 (3)	C10'—C11'—C12'—C13'	0.3 (7)
N2—C1—C7—C8	−32.2 (3)	C11'—C12'—C13'—C14'	0.6 (7)
C39—C1—C7—C6	−158.2 (2)	C12'—C13'—C14'—C15'	−0.8 (7)
C39—C1—C7—C8	88.5 (2)	C13'—C14'—C15'—C10'	0.3 (6)
N2—C1—C7—C6	81.0 (2)	N5'—C16'—C17'—C18'	−179.5 (3)
N2—C1—C39—O40	−121.1 (2)	C21'—C16'—C17'—C18'	0.7 (4)
N2—C1—C39—O43	59.2 (4)	N5'—C16'—C21'—C20'	179.0 (3)
C7—C1—C39—O40	125.5 (2)	C17'—C16'—C21'—C20'	−1.2 (5)
C7—C1—C39—O43	−54.3 (4)	C16'—C17'—C18'—C19'	−0.3 (5)
N2—C3—C8—C7	13.6 (3)	C17'—C18'—C19'—C20'	0.3 (6)
C22—C3—C8—C4	20.6 (3)	C18'—C19'—C20'—C21'	−0.7 (6)
N2—C3—C8—C4	−99.3 (2)	C19'—C20'—C21'—C16'	1.2 (6)
C8—C3—C22—C23	−93.0 (3)	C3'—C22'—C23'—N24'	−169.6 (2)
C8—C3—C22—C26	85.0 (3)	C26'—C22'—C23'—N24'	0.0 (3)
N2—C3—C22—C26	−158.9 (3)	C3'—C22'—C26'—N25'	169.3 (2)
C22—C3—C8—C7	133.6 (2)	C3'—C22'—C26'—C27'	−6.0 (4)
N2—C3—C22—C23	23.2 (4)	C23'—C22'—C26'—N25'	−0.4 (3)
O45—C4—C8—C3	−79.6 (3)	C23'—C22'—C26'—C27'	−175.6 (2)
O45—C4—C8—C7	167.2 (3)	N25'—C26'—C27'—C28'	−46.2 (4)
N5—C4—C8—C3	99.4 (2)	N25'—C26'—C27'—C32'	134.3 (3)
N5—C4—C8—C7	−13.8 (3)	C22'—C26'—C27'—C28'	128.7 (3)
N5—C6—C7—C8	−7.4 (3)	C22'—C26'—C27'—C32'	−50.8 (4)
O44—C6—C7—C1	58.3 (3)	C26'—C27'—C28'—C29'	179.9 (4)
O44—C6—C7—C8	172.6 (2)	C32'—C27'—C28'—C29'	−0.6 (6)
N5—C6—C7—C1	−121.8 (2)	C26'—C27'—C32'—C31'	−177.3 (3)
C1—C7—C8—C3	11.4 (3)	C28'—C27'—C32'—C31'	3.1 (5)
C1—C7—C8—C4	129.6 (2)	C27'—C28'—C29'—C30'	−3.0 (7)
C6—C7—C8—C3	−105.6 (2)	C28'—C29'—C30'—Cl1'	−176.5 (4)
C6—C7—C8—C4	12.6 (2)	C28'—C29'—C30'—C31'	4.2 (7)
N2—C9—C10—C15	29.6 (4)	Cl1'—C30'—C31'—C32'	178.9 (3)
N2—C9—C10—C11	−153.3 (3)	C29'—C30'—C31'—C32'	−1.8 (6)
C15—C10—C11—C12	−0.4 (5)	C30'—C31'—C32'—C27'	−2.0 (6)
C9—C10—C11—C12	−177.7 (3)	N24'—C33'—C34'—C35'	−179.4 (4)
C9—C10—C15—C14	177.1 (3)	C38'—C33'—C34'—C35'	−2.1 (6)
C11—C10—C15—C14	−0.1 (4)	N24'—C33'—C38'—C37'	−179.4 (4)
C10—C11—C12—C13	1.0 (6)	C34'—C33'—C38'—C37'	3.3 (6)
C11—C12—C13—C14	−1.1 (6)	C33'—C34'—C35'—C36'	−1.5 (7)
C12—C13—C14—C15	0.5 (5)	C34'—C35'—C36'—C37'	3.7 (7)
C13—C14—C15—C10	0.1 (5)	C35'—C36'—C37'—C38'	−2.5 (7)
N5—C16—C17—C18	−176.6 (3)	C36'—C37'—C38'—C33'	−1.0 (7)

### Hydrogen-bond geometry (Å, °)

**Table:**

*Cg*1, *Cg*2 and *Cg*3 are the centroids of the C10–C15, C27'–C32' and C33'–C38' rings, respectively.

**Table:**

*D*—H···*A*	*D*—H	H···*A*	*D*···*A*	*D*—H···*A*
C7—H7···O44'	0.98	2.45	3.311 (3)	146
C42'—H42A···Cl1'^i^	0.96	2.76	3.707 (10)	170
C14'—H14'···Cg1	0.93	2.79	3.653 (4)	154
C17'—H17'···Cg2^ii^	0.93	2.95	3.738 (4)	143
C29—H29···Cg1^iii^	0.93	2.87	3.633 (3)	140
C42'—H42B···Cg3^iv^	0.96	2.80	3.866 (9)	153

Symmetry codes: (i) −*x*+1, −*y*+2, −*z*; (ii) −*x*+1, −*y*+1, −*z*; (iii) −*x*+1, −*y*, −*z*+1; (iv) *x*+1, *y*, *z*.
